# Correlating the deep inferior epigastric artery branching pattern with type of abdominal free flap performed in a series of 145 breast reconstruction patients

**DOI:** 10.1308/003588412X13171221592050

**Published:** 2012-10

**Authors:** AR Molina, ME Jones, A Hazari, I Francis, C Nduka

**Affiliations:** Queen Victoria Hospital NHS Foundation Trust,UK

**Keywords:** Breast reconstruction, Free tissue flaps, Surgical flaps, Angiography

## Abstract

**INTRODUCTION:**

The deep inferior epigastric perforator (DIEP) flap is currently viewed as the gold standard in autologous breast reconstruction. We studied three-dimensional computed tomography angiography (CTA) in 145 patients undergoing free abdominal flap breast reconstruction to try to correlate deep inferior epigastric artery (DIEA) branching pattern with the type of flap performed and patient outcome. Today, reconstructive breast surgeons have become more experienced in raising DIEP flaps and operative times are becoming more acceptable. However, there remains significant interest in finding ways to aid this challenging dissection.

**METHODS:**

We retrospectively evaluated consecutive patients between January 2007 and August 2008. CTAs were analysed using the Moon and Taylor (1988) classification of the DIEA branching pattern. Data gathered included pre-operative morbidity, type of abdominal wall free flap performed, length of operation, length of stay and complications.

**RESULTS:**

Some 150 breast reconstructions were performed in 145 patients. There were 67 DIEP flaps, 69 MS-2 transverse rectus abdominis myocutaneous (TRAM) flaps and 14 MS-1 TRAM flaps (where MS-1 spares the lateral muscle and MS-2 spares both lateral and medial segments). Proportionally more DIEP flaps were performed in patients with a type 2 branching pattern. There was one flap loss (0.67%).

**CONCLUSIONS:**

In this large CTA series, we found a type 1 (single artery) DIEA pattern most frequently, in contrast to the predominance of the type 2 bifurcating pattern observed previously. The higher proportion of DIEP flaps performed in the type 2 pattern patients is consistent with the documented shorter intramuscular course in this group. We have found CTA useful for faster selection of the best hemiabdomen for dissection and flap loss rates in our unit have reduced from 1.5% to 0.67%.

There is a growing body of evidence to support deep inferior epigastric perforator (DIEP) flaps as the current gold standard in breast reconstruction. Autologous reconstruction is popular with patients and although funding of the laborious initial procedure is under scrutiny, there are significant long-term advantages. Especially in younger breast reconstruction patients, a totally autologous reconstruction can avoid the need for multiple operations associated with implant longevity and implant associated complications.

The donor site morbidity of free abdominal flap breast reconstruction can be minimised by harvesting a DIEP flap, thereby avoiding the sacrifice of muscle required in the transverse rectus abdominis myocutaneous (TRAM) flap. However, the process of isolating the perforator or perforators was felt initially to add even more time to an already lengthy procedure. Today, reconstructive breast surgeons have become more experienced in raising DIEP flaps and operative times are becoming more acceptable. There is significant interest in finding ways to aid this challenging dissection.

In our unit, computed tomography angiography (CTA) was introduced in late 2006 to attempt to map the course of the abdominal wall perforators. It was postulated that this would aid intra-operative decision making and reduce operating times by focusing the dissection towards the most promising hemiabdomen. Previous studies have in fact shown reduced abdominal free flap operating times following the introduction of CTA^1–3^ and, furthermore, CTA has been shown to be superior to Doppler ultrasonography in mapping the abdominal wall vasculature.^4^

We decided that with the detailed information afforded by three-dimensional CTA, it would be interesting to look at the branching patterns of the deep inferior epigastric artery (DIEA) in our population and categorise them according to Moon and Taylor’s 1988 classification.^5^ This seminal paper grouped the DIEA branching pattern into three types. In 29% of their cadavers, the DIEA did not branch; this was termed type 1. In the majority (57%), the DIEA bifurcated at the arcuate line and this pattern was called type 2. Finally, a trifurcating or type 3 pattern was rarest, observed in only 14% of their cases.

A more recent cadaveric study found that bifurcating type 2 DIEAs were associated with a shorter intramuscular course whereas type 3 DIEAs had the longest intramuscular course.^6^ Type 1 vessels were intermediate. We decided to look back at a large series of abdominal free flap breast reconstruction patients who had undergone pre-operative CTA to evaluate the distribution of the different DIEA branching patterns in our patient population. We also decided to collate data regarding the type of free flap performed (DIEP vs TRAM) to evaluate whether this varied according to the arterial branching pattern.

## Methods

This was a retrospective study of 150 consecutive abdominal free flaps performed in 145 breast reconstruction patients between January 2007 and August 2008. Patients were identified from electronic theatre records and cross-checked against consultant logbooks. The CTA images were reviewed by the radiologists and classified into type 1, 2 or 3 according to the branching pattern of the DIEA. Simultaneously, information was retrieved from case notes including type of flap, length of surgery and inpatient stay as well as any complications.

For CTA of the abdominal wall in our unit, a 32-slice multislice computed tomography scanner is used and 100ml iodinated contrast is injected at 4ml/sec, using SmartPrep software (GE Healthcare, Little Chalfont, UK) to predict the delay. Images are acquired from the level of the diaphragm to the femoral heads at 0.625mm intervals to allow full reconstruction.

## Results

Some 150 breast reconstructions were performed in 145 patients. Of the flaps, 67 were DIEP, 69 were MS-2 TRAM and 14 were MS-1 TRAM flaps (where MS-1 spares the lateral muscle and MS-2 spares both lateral and medial segments, based on the Nahabedian classification).^7^

The mean operating time was 407 minutes for DIEP flaps compared with 490 minutes for TRAM flaps. The mean length of inpatient stay was slightly shorter for DIEP patients at 6.3 days, compared with 7.0 days for TRAM flap patients.

A third of patients had donor site complications such as superficial wound infection, seroma or superficial dehiscence of part of the wound. There were six symptomatic abdominal bulges in TRAM patients. There was one total flap loss, which occurred in a DIEP patient, and one partial flap loss in a MS-2 TRAM case.

**Figure 1:**
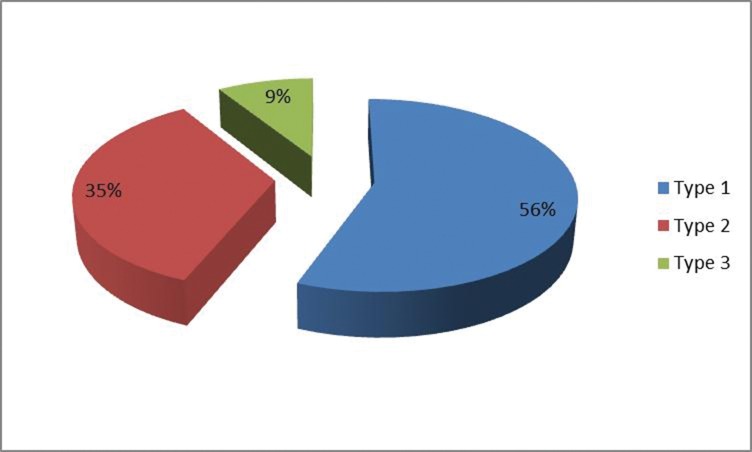
Pie chart showing percentages of DIEA branching patterns observed in our population. The distribution observed was very different from Moon & Taylor’s original paper (in which they found 29% Type 1, 57% Type 2 and 14% Type 3)

**Figure 2:**
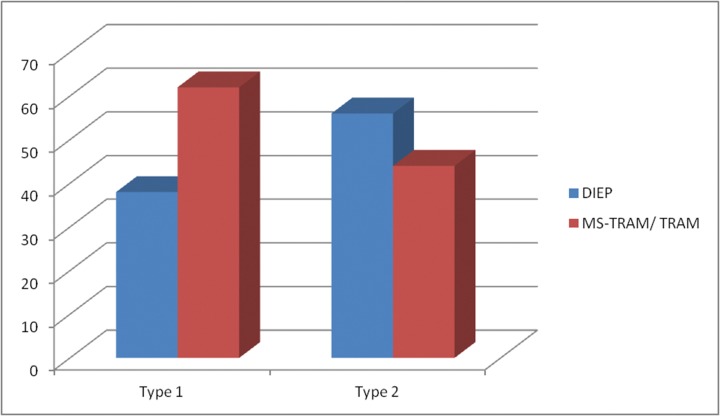
Bar chart showing that more flaps were raised as DIEPs when a Type 2 pattern existed, consistent with the documented shorter intramuscular course for this subset

A type 1 branching pattern was predominant in our patients and was seen in 56% of hemiabdomens. A type 2 (bifurcating) pattern was observed in 35% and the trifurcating type 3 pattern in 9% (Figure 1). Proportionally more DIEP flaps were performed in patients with a type 2 branching pattern: 56% of flaps were raised without muscle sacrifice when a type 2 pattern existed, compared with only 38% raised as DIEP flaps in the presence of a type 1 branching pattern (Figure 2).

## Discussion

Operative times in our patient series were found to be lowest in DIEP and highest in TRAM flaps. We suggest that the longer operative time recorded for muscle sparing TRAM flaps is because surgeons spend time considering whether muscle sacrifice is indeed essential. It would be interesting to compare mean operative times now with those prior to the introduction of CTA in our unit. Inpatient stay was found to be slightly shorter for DIEP patients compared with TRAM patients, in keeping with the established literature.

It is encouraging that there was just one flap loss in this study. Previous audits in our institution have generated flap loss rates of around 1.5%, in contrast to just 0.67% for this group. Other reports have documented reduced complication rates with the introduction of CTA.^1^ There were, however, six symptomatic abdominal bulges or hernias in our patients, which were mostly observed towards the start of the study. It has since become standard practice to use mesh in all our MS-TRAM patients and often even in DIEP patients. We believe this has led to decreased donor site morbidity although we are awaiting a longer follow-up period to justify this theory.

The overall feedback about CTA in our unit is very positive, including its advantages for training (allowing the trainee to be ‘talked through’ the dissection pre-operatively using the scans, ideally in conjunction with an experienced radiologist). Consultants can select those flaps that are predicted to have a straightforward dissection for trainees to raise. Following a detailed examination of the imaging, a decision is made regarding which side to raise the flap on and whether medial or lateral perforators are most suitable. The patient can be counselled pre-operatively about possible donor site morbidity if the perforators have a long intramuscular course and muscle sacrifice appears likely to be required. It is of course always prudent to have a fallback plan (or preferably two) in case perforators are found to be of inadequate size or are inadvertently damaged intra-operatively.

We wanted to highlight that in addition to the obvious advantages of CTA, it can also reveal contraindications to surgery that would not otherwise have been apparent.^8^ Three patients in our unit were excluded from free TRAM/DIEP flaps as a result of incidental CTA findings, eg for severe arterial occlusive disease or incidental malignancy. Other groups have reported the value of CTA in determining when not to operate or when to amend the operative plan.^9^

## Conclusions

We showed a different distribution of DIEA branching patterns in our study to that documented previously; fewer type 2 patterns were observed and, instead, type 1 DIEAs were predominant. We had postulated that this difference could be geographical as Moon and Taylor’s study was in the Australian population. However, since our data was collected a further in vivo Australian study of DIEA branching patterns in 500 hemiabdomens has been published.^10^ The results appear to corroborate our findings: the researchers found a higher proportion of type 1 and a lower proportion of type 3 patterns than expected compared with Moon and Taylor’s 1988 cadaveric study.

Our results also support the previous finding that the bifurcating (type 2) DIEA pattern has the shortest intramuscular course^6^ as we were able to perform more DIEP flaps when this pattern existed. We would like to emphasise that we are a pragmatic unit and that CTA findings should be considered in context with other factors when making operative decisions. CTA is, however, proving to be a very useful addition to our armamentarium and new developments such as navigation systems^11^ may help to further enhance its role in pre-operative planning.
